# The Cat Flea (Ctenocephalides felis) Immune Deficiency Signaling Pathway Regulates Rickettsia typhi Infection

**DOI:** 10.1128/IAI.00562-17

**Published:** 2017-12-19

**Authors:** Sherri A. Rennoll, Kristen E. Rennoll-Bankert, Mark L. Guillotte, Stephanie S. Lehman, Timothy P. Driscoll, Magda Beier-Sexton, M. Sayeedur Rahman, Joseph J. Gillespie, Abdu F. Azad

**Affiliations:** a Department of Microbiology and Immunology, University of Maryland School of Medicine, Baltimore, Maryland, USA; b Department of Biology, West Virginia University, Morgantown, West Virginia, USA; Yale University School of Medicine

**Keywords:** Ctenocephalides felis, IMD, Rickettsia typhi, flea, innate immunity, vector biology

## Abstract

Rickettsia species are obligate intracellular bacteria with both conserved and lineage-specific strategies for invading and surviving within eukaryotic cells. One variable component of Rickettsia biology involves arthropod vectors: for instance, typhus group rickettsiae are principally vectored by insects (i.e., lice and fleas), whereas spotted fever group rickettsiae are exclusively vectored by ticks. For flea-borne Rickettsia typhi, the etiological agent of murine typhus, research on vertebrate host biology is facilitated using cell lines and animal models. However, due to the lack of any stable flea cell line or a published flea genome sequence, little is known regarding R. typhi biology in flea vectors that, importantly, do not suffer lethality due to R. typhi infection. To address if fleas combat rickettsial infection, we characterized the cat flea (Ctenocephalides felis) innate immune response to R. typhi. Initially, we determined that R. typhi infects Drosophila cells and increases antimicrobial peptide (AMP) gene expression, indicating immune pathway activation. While bioinformatics analysis of the C. felis transcriptome identified homologs to all of the Drosophila immune deficiency (IMD) and Toll pathway components, an AMP gene expression profile in Drosophila cells indicated IMD pathway activation upon rickettsial infection. Accordingly, we assessed R. typhi-mediated flea IMD pathway activation *in vivo* using small interfering RNA (siRNA)-mediated knockdown. Knockdown of *Relish* and *Imd* increased R. typhi infection levels, implicating the IMD pathway as a critical regulator of R. typhi burden in C. felis. These data suggest that targeting the IMD pathway could minimize the spread of R. typhi, and potentially other human pathogens, vectored by fleas.

## INTRODUCTION

Globally, vector-borne pathogens account for approximately 17% of infectious diseases and cause over 700,000 deaths each year (1). Several factors, such as climate change and the globalization of trade, contribute to the emergence and reemergence of vector-borne diseases throughout the world (2, 3). While approaches to control vector population through insecticide use have proven successful, vector resistance limits their continued effectiveness (4). The design and implementation of innovate prevention programs require a thorough understanding of the vector response to pathogens. One emergent method to control the spread of vector-borne pathogens is genetic modification of the vector immune response to prevent pathogen survival and transmission. Such an approach requires in-depth knowledge of the host's immune system, which for arthropod vectors is becoming increasingly more realized (5).

Vector biology research is predominately focused on mosquitoes and ticks, transmitters of many serious human pathogens (6–8). Fleas are among many other underappreciated vectors, despite transmitting several human pathogens, including Yersinia pestis, Rickettsia typhi, Rickettsia felis, and Bartonella species (9, 10). As climate change and destruction of habitats drive dramatic alterations in the geographic and host ranges of fleas, flea-borne diseases are emerging and reemerging throughout the world, as exemplified by the reemergence of plague (caused by Y. pestis) in Africa, South America, and the western United States (11) and R. typhi, the etiological agent of murine typhus, in southern Texas and California (12–15).

The intermittent blood feeding behavior of fleas, practically on all warm-blooded hosts, allows them to potentially become vectors for any pathogen found in a blood meal, including viruses, which were previously demonstrated to be transmitted by fleas in the laboratory setting (16). Thus, it is imperative that the factors making the flea a competent vector for human pathogens be identified. Research efforts, however, are thwarted by the lack of a sequenced flea genome or a flea cell line. Despite this, it has been demonstrated that upon taking a blood meal, the cat flea, Ctenocephalides felis, upregulates the expression of genes associated with immune defense (17). Pathogen evasion of this antibacterial response is likely required for optimal vector colonization and disease transmission. In support of this, we previously demonstrated that nine candidate flea antimicrobial genes (those for defensin A, serpins 4, 5, and 7, complement component 1q binding-like protein, PGRP-LB, two galactose-specific C-type lectins, and tetraspanin) were differentially regulated in C. felis midguts during infection with R. typhi (18), indicating that R. typhi is eliciting a flea immune response. Thus, characterizing flea innate immune pathways may illuminate targets for prevention of pathogen transmission.

While all species of Rickettsia are obligate intracellular bacteria that invade and survive within eukaryotic cells, host range is variable across different species (19). For instance, the three derived Rickettsia lineages include species with different principal vectors: typhus group (lice and fleas), transitional group (mites, ticks, and fleas) and spotted fever group (ticks) (20). As a species of typhus group rickettsiae, R. typhi is principally vectored by two human biting fleas, C. felis and the rat flea, Xenopsylla cheopis, and thus serves as a model to illuminate the mechanisms by which fleas control pathogen infection. R. typhi is typically maintained in a natural cycle with small rodents and fleas (21). Once ingested with an infected blood meal, R. typhi enters the flea midgut lumen and infects epithelial cells within the first 30 min. After principally colonizing the entire midgut epithelium, R. typhi enters the hemocoel and induces systemic infection (22–24). As we have shown previously, R. typhi infection in fleas causes no observable impact on flea fitness and does not shorten the adult flea life span under experimental conditions (12, 25, 26). The ability of infected fleas to cope with the persisting infection provides continuous pathogen transmission. Despite this knowledge, the molecular mechanisms underpinning the flea's response to R. typhi infection are not yet known.

In this work, we overcame the lack of an available flea cell line by demonstrating that R. typhi successfully grows in Drosophila melanogaster cell lines and triggers the immune deficiency (IMD) signaling pathway, the typical insect immune response to Gram-negative pathogens (27, 28). Furthermore, in circumventing the lack of a C. felis genome sequence, bioinformatics analysis of the C. felis transcriptome identified homologs to all of the Drosophila IMD pathway components, allowing for the design of antisense RNAs to genes encoding these components. The successful knockdown of these targets in *C. felis in vivo* increased R. typhi infection load, implicating the IMD signaling pathway as a critical regulator of R. typhi burden in the flea. Thus, targeting the IMD pathway or the innate immune response in the flea could diminish or prevent the transmission of R. typhi, as well as other flea-borne pathogens.

## RESULTS

### R. typhi infects Drosophila cells.

**FIG 1:**
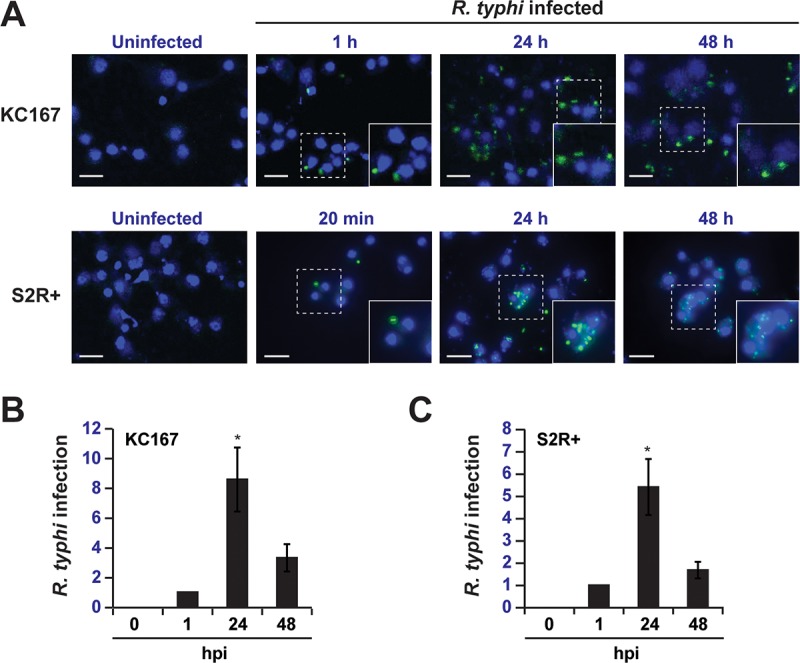
R. typhi infects Drosophila cell lines. (A) Indirect immunofluorescence analysis of KC167 and S2R+ cells infected with R. typhi. Cells were immunostained with anti-R. typhi serum (green) at 0 min, 20 min, 1 h, 24 h, and 48 h postinfection. Nuclei were counterstained with DAPI (blue). (B and C) Analysis of R. typhi infection in KC167 (B) and S2R+ (C) cells 0, 1, 24, and 48 h postinfection (hpi) by RT-qPCR. Data were normalized to the housekeeping gene *Actin* and 1 h postinfection. Error bars indicate SEMs of three independent experiments conducted in duplicate. *, *P* < 0.05 (relative to 1 hpi; Student's two-sided *t* test).

Experiments to address activation of the flea innate immune response by R. typhi are inherently difficult due to the lack of a flea cell line and sequenced flea genome. Because the immune system is well characterized and a plethora of genetic tools are available for analyzing gene function (29, 30), we reasoned that Drosophila cells could serve as a model to initially characterize the vector immune response to R. typhi. Hemocytes are cells that circulate freely in the hemolymph and are able to synthesize and secrete antimicrobial peptides (AMPs). We therefore tested whether R. typhi could infect the Drosophila hemocyte-like KC167 and S2R+ cell lines, which can elicit an immune response upon microbial infection and induce AMP gene expression (31). Using anti-R. typhi serum to immunostain KC167 and S2R+ cells treated with R. typhi, we detected R. typhi in KC167 cells at 1, 24, and 48 h postinfection and in S2R+ cells at 20 min, 24 h, and 48 h postinfection (Fig. 1A). We also measured R. typhi infection in KC167 and S2R+ cells using quantitative reverse transcription-PCR (RT-qPCR) (Fig. 1B and C). R. typhi was not detected in either cell line at 0 h postinfection but was readily detected 1, 24, and 48 h postinfection. A significant increase in R. typhi infection was observed between 1 and 24 h postinfection, followed by a decrease in R. typhi between 24 and 48 h postinfection for both KC167 and S2R+ cells. Together, these results indicate that R. typhi is able to infect Drosophila KC167 and S2R+ cells and that these cell lines could serve as a model for studying activation of flea innate immunity by R. typhi.

### R. typhi elicits an immune response in Drosophila cells.

**FIG 2:**
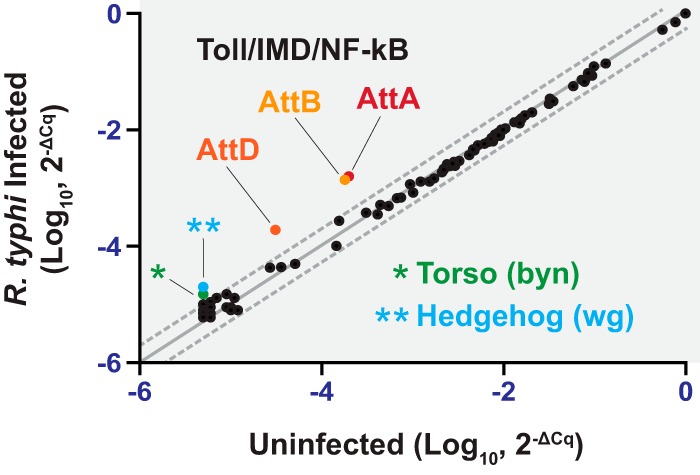
R. typhi elicits an immune response in Drosophila cells. Shown are results of a PCR array analysis of transcripts in uninfected and R. typhi-infected KC167 cells. RNA was extracted from KC167 cells infected with R. typhi for 0 or 4 h, and cDNA was used in the Fruit Fly Signal Transduction PathwayFinder PCR array.

Upon infection with a microorganism, immune signaling pathways are activated in Drosophila cell lines (32). To determine whether an immune response is elicited in Drosophila cells after infection with R. typhi, we infected KC167 cells with rickettsiae and monitored changes in gene expression at 4 h postinfection using a preformatted gene pathway array. Using this PCR array, we compared expression levels of 84 genes representing 12 signaling pathways between uninfected and R. typhi-infected KC167 cells. We identified five genes that were significantly upregulated in response to R. typhi infection but did not identify any significantly downregulated genes (Fig. 2). The five genes with increased expression in response to R. typhi represented three pathways on the PCR array: (i) Hedgehog (*wg*), (ii) Torso (*byn*), and (iii) Toll/IMD/NF-κB (*AttA*, *AttB*, and *AttD*). The Attacin genes (*AttA*, *AttB*, and *AttD*) encode AMPs and their increased expression indicates activation of immune signaling in R. typhi-infected KC167 cells. Increased expression of other immune genes representing the Toll/IMD/NF-κB pathways, including Cactus, Defensin, Drosomycin, and Metchnikowin, were not observed with the PCR array.

**FIG 3:**
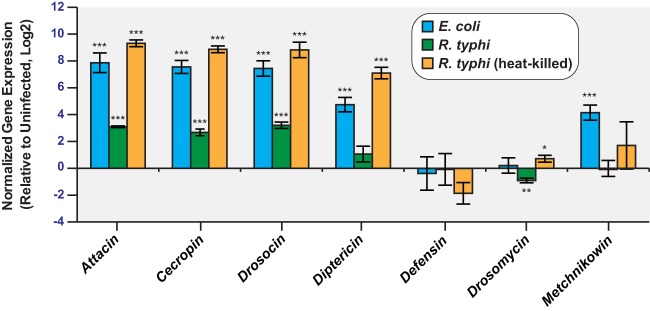
AMP transcripts are elevated in R. typhi-infected KC167 cells. Shown are results of an RT-qPCR analysis of AMP gene expression in cDNAs prepared from KC167 cells. Transcripts were measured in uninfected cells and in cells infected with E. coli or R. typhi for 4 h or in cells treated with heat-killed R. typhi for 4 h. The data are normalized to the housekeeping genes *Actin* and *Rpl32* and the uninfected control. Error bars indicate SEMs. *, *P* < 0.05; **, *P* < 0.01; ***, *P* < 0.001 (relative to uninfected cells; Student's two-sided *t* test).

To further confirm and expand upon the PCR array results, we measured transcript levels of seven AMP genes in uninfected and R. typhi-infected KC167 cells (Fig. 3). Similar to the case with the PCR array, Attacin gene expression was increased, but no significant increase in Defensin, Drosomycin, or Metchnikowin transcript levels was observed. Transcript analysis of Cecropin and Drosocin revealed that the expression of these two genes was increased in response to R. typhi infection, but no significant increase was observed for Diptericin. The observed AMP gene expression changes in R. typhi-infected KC167 cells followed a pattern similar to that of KC167 cells infected with the Gram-negative bacterium Escherichia coli, which is known to induce an immune response in Drosophila cells (33). KC167 cells infected with R. typhi and cells treated with heat-killed R. typhi displayed similar patterns of gene expression; however, greater increases in AMP gene expression were observed for KC167 cells treated with heat-killed R. typhi. Together, these results demonstrate that infection of KC167 cells with R. typhi induces innate immune signaling and expression of a subset of AMP genes.

### IMD and Toll signaling pathways are conserved between Drosophila and C. felis.

**FIG 4:**
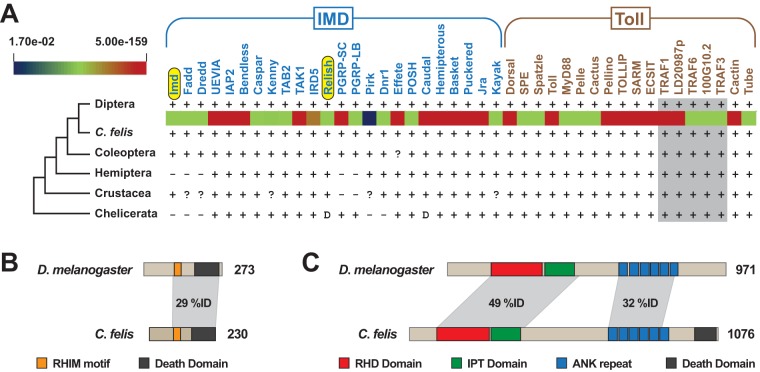
IMD and Toll signaling pathways are conserved between Drosophila and C. felis. (A) C. felis contains homologs to D. melanogaster IMD (*n* = 24) and Toll (*n* = 18) pathway genes. D. melanogaster genes were used as queries in reciprocal best blastn searches against the C. felis 1KITE transcripts. E-values for matches are depicted by a heat map between C. felis and Diptera (at the top is the range of E values across all significant hits). The distribution of these genes in other arthropod genomes is shown as follows: −, absent from lineage; ?, uncertain homology; and D, divergent architecture. Gray shading indicates that tumor necrosis factor receptor-associated factor (TRAF) homologies were not confidently assigned outside the C. felis/D. melanogaster comparisons. (B and C) Comparison of Drosophila and C. felis proteins encoded by *Imd* (B) and *Relish* (C). C. felis proteins were translated from transcripts identified by tblastn searches against the C. felis 1KITE transcripts using the proteins encoded by D. melanogaster *Imd* (NCBI accession no. NP_573394) and *Rel* (NP_477094). Protein domains were predicted using the SMART database (54): RHD, Rel homology DNA binding; IPT, ig-like, plexins, transcription factors; ANK, ankyrin. The Rip homotypic interaction (RHIM) motif was demarcated as previously characterized (53).

Activation of both the IMD and Toll signaling pathways can lead to induction of AMP gene expression. To date, neither of these pathways has been characterized for C. felis. To gain a better understanding of the genetic makeup of the C. felis IMD and Toll pathways, we mined the C. felis 1KITE transcripts (34) for the presence of IMD and Toll pathway genes, using Drosophila gene sequences as queries. Remarkably, all 42 Drosophila genes were found to have counterparts within the C. felis genome (Fig. 4A). Domain analysis of the translated C. felis Imd and Rel proteins indicated conservation with D. melanogaster Imd and Rel proteins (Fig. 4B and C). Not only does this reveal the makeup of C. felis IMD and Toll pathways, but it also strengthens the conclusion that Drosophila cells are a valid system for *in vitro* work given the lack of a flea cell line.

### The IMD signaling pathway controls R. typhi burden in C. felis.

**FIG 5:**
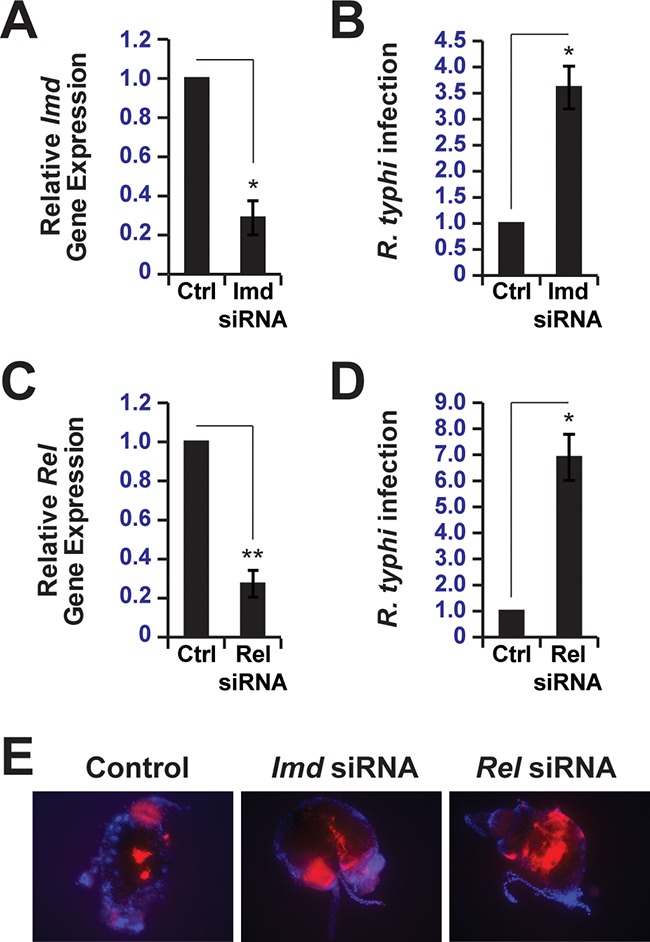
The IMD signaling pathway controls R. typhi burden in C. felis. (A) RT-qPCR analysis of *Imd* transcripts from fleas fed blood meals containing control (Ctrl) or *Imd* siRNAs. Data were normalized to the housekeeping genes *Actin* and *Ef* and fleas fed blood meals containing control siRNAs. Error bars indicate SEMs. *, *P* < 0.05. (B) Analysis of R. typhi infection in fleas fed blood meals containing control or *Imd* siRNAs by RT-qPCR. Data were normalized to the housekeeping genes *Actin* and *Ef* and fleas fed blood meals containing control siRNAs. Error bars indicate SEMs. *, *P* < 0.05. (C) Same as in panel A except that *Rel* transcripts from fleas fed blood meals containing control or *Rel* siRNAs were analyzed by RT-qPCR. Error bars indicate SEMs. **, *P* < 0.01. (D) Same as in panel B except that *Rel* siRNAs were used in blood meals instead of *Imd* siRNAs. Error bars indicate SEMs. *, *P* < 0.05. (E) Representative images of mCherry-R. typhi (red) infection in isolated flea midguts. Fleas were fed blood meals containing control, *Imd*, or *Rel* siRNAs and mounted on slides using ProLong Gold antifade mountant with DAPI (blue). Separate images were obtained using DAPI and TRITC filter sets and merged to generate the composite images shown.

Our *in vitro* results using Drosophila KC167 cells indicated an insect innate immune response to R. typhi, thus providing a rationale for *in vivo* experiments using adult cat fleas. As our data demonstrate that both the IMD and Toll pathways are conserved between Drosophila and C. felis (Fig. 4), we focused our attention on the contribution of the IMD pathway to R. typhi burden in the cat flea. The rationale for pursuing the IMD pathway is that Drosocin, an IMD-specific target gene, but not Drosomycin, a target of the Toll pathway, was upregulated in KC167 cells infected with R. typhi (Fig. 3) (32, 35–37). To investigate the contribution of the IMD pathway to R. typhi burden in C. felis, we knocked down *Imd* (encoding immune deficiency, isoform A) or *Relish* (*Rel*, encoding the nuclear factor NF-κB p110 subunit), components of the IMD pathway, and measured R. typhi infection. Using RT-qPCR, we detected decreased *Imd* and *Rel* transcript levels in the midguts of fleas fed blood meals containing *Imd* or *Rel* small interfering RNAs (siRNAs), respectively, relative to those in fleas fed blood meals containing control siRNAs (Fig. 5A and C). We predicted that the observed decrease in *Imd* and *Rel* transcripts would reduce nuclear Relish levels and the expression of target genes, including those for AMPs, thus preventing the flea from activating a full immune response against R. typhi. Indeed, decreased *Imd* and *Rel* transcript levels corresponded to an increase in R. typhi infection, as measured by RT-qPCR (Fig. 5B and D). Increased R. typhi burden in *Imd* and *Rel* knockdown fleas relative to that in control fleas was also observed by immunofluorescence microscopy of flea midguts (Fig. 5E). The increase in R. typhi burden in *Imd* and *Rel* knockdown fleas relative to that in control fleas indicates that the IMD pathway controls R. typhi burden in the cat flea.

## DISCUSSION

The vector innate immune response plays a key role in the spread and transmission of vector-borne diseases (5). While insects respond to microbial infection using similar immune response mechanisms, including the secretion of AMPs and production of reactive oxygen species, their susceptibilities to various microbes differ (38). Such variation in vector susceptibility is partially attributed to the immunity of the vector (5). While the vector immune response plays a critical role in susceptibility to a particular microbe, we know very little about how fleas respond to microbial infection. This can largely be attributed to the lack of a flea cell line and sequenced flea genome. In this study, we modeled R. typhi infection *in vitro* using Drosophila cells and then expanded our findings to the cat flea *in vivo*. By taking this approach, we demonstrate that Drosophila cells are a potential model for studying the innate immune response to R. typhi and that the IMD signaling pathway is critical for controlling R. typhi burden in the flea.

We demonstrated that rickettsiae grow well in Drosophila cells (Fig. 1) and that immune signaling pathways within these cells are activated by R. typhi, as evidenced by increased AMP gene expression (Fig. 2 and 3). Interestingly, R. typhi activated AMP gene expression to a lesser extent than heat-killed R. typhi. This could indicate that upon heat killing of R. typhi, immune reactive components that are normally hidden are now exposed (5), resulting in a greater immune response and increased AMP gene expression. Conversely, it is also possible that R. typhi actively suppresses the Drosophila immune response to establish infection. This phenomenon has been described for both Pseudomonas entomophila and Pseudomonas aeruginosa. AprA, a zinc metalloprotease, is secreted by P. entomophila and provides protection against the AMP diptericin (39). Similarly, P. aeruginosa represses AMP gene expression in Drosophila melanogaster, but the mechanism used to do so is not yet known (40). As our results identify Drosophila cells as a potential model for studying the vector immune response to R. typhi, these cells could be used to address whether R. typhi is able to repress AMP gene expression and, therefore, suppress the fly immune response.

Analysis of AMP gene expression changes in Drosophila KC167 cells indicated increased Attacin, Cecropin, and Drosocin transcript levels (Fig. 2 and 3). No significant increase in Diptericin, Defensin, Drosomycin, and Metchnikowin gene expression was observed. Attacin, Cecropin, Drosocin, and Diptericin are generally viewed as targets of the IMD pathway, while Drosomycin is viewed as a target of the Toll pathway (32, 35–37). It has become apparent, however, that the IMD and Toll pathways activate many of the same target genes, including Attacin, Cecropin, Diptericin, Drosomycin, and Metchnikowin (41, 42). For the work described here, we focused our attention on the IMD pathway in the flea because R. typhi is a Gram-negative bacterium and we observed an increase in Drosocin gene expression. However, the increase in Attacin and Cecropin gene expression may indicate that the C. felis Toll pathway, which is highly homologous to the dipteran Toll pathway, contributes to the immune response against R. typhi. Future work will address this possibility.

By feeding fleas blood meals containing control, *Imd*, or *Rel* siRNAs, we were able to knock down *Imd* and *Rel* transcripts in the flea (Fig. 5). Decreased expression of *Imd* and *Rel* corresponded to increased R. typhi burden in the flea, demonstrating that these two IMD pathway components are critical for mounting an effective immune response against R. typhi. Interestingly, despite similar levels of knockdown between *Imd* and *Rel* transcripts, knockdown of *Imd* increased R. typhi infection in the flea approximately 3.5-fold, while *Rel* knockdown increased R. typhi infection approximately 7-fold. It is feasible that the observed differences in R. typhi infection can be attributed to the amount of knockdown at the protein level. However, it is also possible that the observed differences are a result of the upstream and downstream locations of these proteins within the IMD pathway (43). Relish acts as the downstream effector of the IMD pathway by directly regulating target gene expression (44, 45). We expect AMP gene activation to be severely inhibited by knocking down *Rel*, preventing the flea from mounting an effective immune response. We hypothesize that the increase in R. typhi burden upon *Imd* knockdown occurs through a manner similar to that for *Rel* knockdown. However, because Imd is an upstream activator of the pathway, it is possible that components from other signaling pathways compensate for its loss (43), affording a more effective immune response than that in fleas with *Rel* knocked down. Unfortunately, due to the lack of a sequenced flea genome, it is difficult to address how AMP gene expression is affected by the knockdown of *Imd* and *Rel*. While preexisting transcriptome data for the flea (34) could be used to identify putative AMP genes based on Drosophila sequences, many AMP genes would likely remain to be identified. This is due to the fact that AMP gene expression is activated in response to immune pathway signaling, but the transcripts sequenced to generate the flea transcriptome were not from fleas induced to have active immune signaling. Therefore, to fully characterize the flea immune response to R. typhi and other pathogens, it is critical that flea genome sequences become available.

We predict that diminished IMD signaling, as a result of *Imd* and *Rel* knockdown, led to increased R. typhi burden in the flea. Therefore, the ability of the IMD pathway to control R. typhi infection in the flea indicates that the flea innate immune response is an ideal therapeutic target for preventing the spread of R. typhi. Importantly, therapeutics that act by increasing flea immune signaling or inhibiting R. typhi, or other microbes, from suppressing the immune response could be used to prevent disease transmission by fleas.

## MATERIALS AND METHODS

### Bacterial strains, cell culture, and infection.

KC167 and S2R+ cells (generous gifts from Dana Shaw at the University of Maryland Baltimore and Neal Silverman at University of Massachusetts, respectively) were maintained in Schneider's medium (Life Technologies; 21720-024) with 10% heat-inactivated fetal bovine serum (FBS; Thermo Fisher; 10438-026) at 28°C. Vero76 cells (ATCC; CRL-1587) were maintained in minimal Dulbecco's modified Eagle's medium (DMEM) with 4.5 g/liter of glucose and l-glutamine (Corning; 10-017-CM) supplemented with 10% heat-inactivated FBS (Gemini; 100-500) at 37°C with 5% CO_2_. R. typhi strain Wilmington (ATCC; VR-144) was propagated in Vero76 cells grown in DMEM supplemented with 5% heat-inactivated FBS at 34°C with 5% CO_2_. Rickettsiae were partially purified as previously described (46).

### Transformation.

Purified R. typhi cells were transformed with pRAMF2-mCherry (a modified version of the pRAM18dRGA plasmid [47] with the gene encoding mCherry) (N. Noriea and T. Hackstadt, unpublished data) by following previous approaches for transforming rickettsiae (48–50), but with several adjustments. Specifically, about 10^9^ to 10^10^ rickettsiae were Renografin purified, electroporated with 10 μg of pRAMF2-mCherry, and added onto Vero cell monolayers for 30 min at 37°C to achieve infection. After 24 h, medium was changed to DMEM with 5% FBS and 200 ng/ml of rifampin. The infection was allowed to progress until plaque formation was observed (approximately 14 days). Clonal transformants were obtained by three repetitions of picking individual plaques and expanding the plaques by limiting dilution in Vero cell monolayers with DMEM containing 5% FBS and 200 ng/ml of rifampin. Transformants (mCherry-R. typhi) were verified by immunofluorescence analysis and PCR.

### Immunofluorescence.

Following overnight pretreatment with 1 μM 20-hydroxyecdysone (Sigma; H5142), a steroid hormone that enhances the innate immune response in Drosophila cells (51, 52), KC167 and S2R+ cells were infected with R. typhi at a multiplicity of infection (MOI) of ∼100:1. Uninfected and wild-type R. typhi-infected KC167 and S2R+ cells pretreated with 20-hydroxyecdysone were subsequently washed three times with phosphate-buffered saline (PBS) and fixed with 4% paraformaldehyde (PFA) for 10 min at room temperature. Following three washes with PBS, the cells were permeabilized in blocking buffer (0.3% saponin and 0.5% goat serum in PBS) for 30 min and then incubated for 1 h with rat anti-R. typhi serum diluted 1:500 in antibody dilution buffer (0.3% saponin in PBS). The cells were subsequently washed three times with PBS and incubated with anti-rat Alexa Fluor 488 secondary antibodies (Thermo Fisher Scientific; A21208) diluted 1:1500 in antibody dilution buffer for 1 h. After three washes with PBS, the slides were mounted using ProLong Gold antifade mountant with 4′,6-diamidino-2-phenylindole (DAPI) (Thermo Fisher Scientific; P36931) and visualized using a Zeiss LSM510 Meta of LSM5 DUO confocal microscope (University of Maryland Baltimore Confocal Core Facility).

### PCR array.

KC167 cells were treated with 1 μM 20-hydroxyecdysone overnight and left uninfected or infected with E. coli strain MC4100, wild-type R. typhi, or wild-type R. typhi heat killed at 95°C for 30 min at an MOI of ∼100:1. Four hours postinfection, RNA was isolated from KC167 cells using the Quick-RNA Mini-Prep Plus kit (Zymo Research; R1055) and cDNAs were synthesized from 200 ng of RNA using an RT^2^ First Strand kit (Qiagen; 330401). Synthesized cDNA samples were analyzed by quantitative real-time PCR (qPCR) using a Stratagene Mx3000P qPCR system and a RT^2^ Profiler Fruit Fly Signal Transduction PathwayFinder PCR array (Qiagen; PADM-014Z) according to the manufacturer's instructions. Data (see Table S3 in the supplemental material) were analyzed using RT^2^ profiler PCR Array Data Analysis software, version 3.5 (Qiagen), available online at http://pcrdataanalysis.sabiosciences.com/pcr/arrayanalysis.php.

### Quantitative reverse transcription-PCR.

After overnight pretreatment with 1 μM 20-hydroxyecdysone, KC167 and S2R+ cells were infected with R. typhi at an MOI of ∼100:1. RNA was isolated 0, 1, 4, 24, or 48 h postinfection using the Quick-RNA miniprep kit (Zymo Research; R1055). The iScript Reverse Transcription Supermix kit (Bio-Rad; 1708841) was used to synthesize cDNAs from 200 ng of RNA according to the manufacturer's instructions. Transcript levels were assessed by diluting cDNA samples 1:1.5 and using 1 μl in 10-μl qPCR mixtures containing 2× VeriQuest SYBR green master mix (Affymetrix; 75600) and 10 pmol each of forward and reverse primers (see Table S1). The reactions were cycled at 50°C for 2 min and 95°C for 10 min, followed by 40 cycles of 95°C for 15 s and 55°C for 30 s. A melt curve analysis was then performed using the Bio-Rad CFX384 real-time system. Relative R. typhi *ompB* gene expression was measured using the comparative threshold cycle (2^−ΔΔCT^) method with *Actin* as the reference transcript and 1 h postinfection as the calibrator for R. typhi infection in KC167 and S2R+ cells. For AMP gene expression analysis in KC167 cells, the 2^−ΔΔCT^ method was also used to measure relative expression with *Actin* and *Rpl32* as reference transcripts and the uninfected control as the calibrator.

### Bioinformatics analysis.

To determine the composition of the C. felis innate immune pathways, D. melanogaster IMD (*n* = 24) and Toll (*n* = 18) pathway genes (Fig. 4) were used as queries in reciprocal best blastn searches (E-value < 0.1) against the C. felis 1KITE transcripts (34). Comparative analyses of the IMD and Toll pathways across several arthropod groups were then made to identify the taxon with the immune gene profile most similar to that of C. felis. Within the reconstructed C. felis IMD pathway, two genes were selected for further *in silico* characterization: *Imd* (encoding immune deficiency, isoform A) and *Relish* (*Rel*, encoding the nuclear factor NF-κB p110 subunit). Protein domains were predicted using the SMART database, with an additional motif within Imd proteins, the Rip homotypic interaction (RHIM) motif, demarcated as previously characterized (53). The sequences of the *Imd* (NCBI accession number GAYP02008970) and *Rel* (GAYP02012752) transcripts were subsequently used to select regions for designing antisense RNAs for use in gene knockdown assays described below.

### Flea siRNA treatment and infection.

Approximately 50 to 100 fleas (HESKA Corp., Loveland, CO) per treatment group were prefed defibrinated sheep's blood (Cleveland Scientific, Bath, OH) for 72 h, followed by blood deprivation for 5 h. Fleas were then fed an siRNA-containing blood meal for 16 h. The siRNA blood meals were prepared by adding 500 μl of defibrinated sheep's blood to individual siRNA reaction mixtures that were prepared by incubating 1000 pmol of three individual siRNAs (Table S2) with 24 μl of Escort IV transfection reagent (Sigma; L3287) for 15 min at room temperature. For the negative-control reaction, 1,000 pmol of a scrambled siRNA was used. Following the siRNA blood meal, fleas were fed on regular blood for 24 h and then deprived of blood for 5 h. After blood deprivation, fleas were simultaneously treated with siRNA a second time and infected with mCherry-R. typhi. The infected siRNA blood meal was prepared by adding approximately 8 × 10^8^ partially purified rickettsiae to the siRNA blood meal described above. Following 16 h of feeding on the infected siRNA blood meal, fleas were fed on regular blood for the duration of the experiment. Four and 10 days after the initial siRNA treatment, midguts isolated from 10 to 15 fleas were homogenized in 300 μl of RNA lysis buffer (Zymo Research; R1060-1-50) and RNA was isolated using a Quick-RNA miniprep kit (Zymo Research; R1055). cDNA synthesis and qPCR were subsequently performed as described above, except that R. typhi *ompB* and *adr1* transcripts were used to measure relative R. typhi infection using the 2^−ΔΔCT^ method with *Actin* and elongation factor 1α (*Ef*) as the reference transcripts and the control siRNA sample as the calibrator. Additionally, flea midguts were isolated and mounted on glass slides using ProLong Gold antifade mountant with DAPI. The slides were visualized using a Nikon Eclipse E600 microscope. Images obtained using DAPI and tetramethylrhodamine (TRITC) filter sets were merged using Adobe Photoshop to create the final composite images.

### Statistical analysis.

Each experiment was repeated at least three times, and Student's two-sided *t* test was used to calculate statistical significance.

## Supplementary Material

Supplemental material
