# Supplementary material for: The Cat Flea (Ctenocephalides felis) Immune Deficiency Signaling Pathway Regulates Rickettsia typhi Infection
Source: Infect Immun. 2017 Dec 19;86(1):e00562-17. doi: 10.1128/IAI.00562-17 (PMC5736803; doi:10.1128/IAI.00562-17)
Supplement: Supplemental material [file supp_86_1_e00562-17__index.html]

Supplemental material 

# The Cat Flea (Ctenocephalides felis) Immune Deficiency Signaling Pathway Regulates Rickettsia typhi Infection

## Supplemental material

- Supplemental file 1 -

  Table S1. Oligonucleotide sequences for RT-qPCR. Table S2. siRNA sequences for knockdown in *C. felis*. Table S3. Raw data used for analysis of the PCR array conducted in uninfected (Vero 1-5) and *R. typhi*-infected (RT 1-5) KC167 cells.

  PDF, 278K
