# Supplementary material for: The Cat Flea (Ctenocephalides felis) Immune Deficiency Signaling Pathway Regulates Rickettsia typhi Infection
Source: Infect Immun. 2017 Dec 19;86(1):e00562-17. doi: 10.1128/IAI.00562-17 (PMC5736803; doi:10.1128/IAI.00562-17)
Supplement: Supplemental material [file IAI.00562-17_zii999092265s1.pdf]

Table S1 Oligonucleotide sequences for RT-qPCR.

**AMP gene expression in KC167 cells**		
**Oligonucleotide name**	**Oligonucleotide sequence**	**NCBI accession number**
dmAttachin_F	GGCCCATGCCAATTTATTCA	NM_001169681.2
dmAttacin_R	CATTGCGCTGGAACTCGAA	NM_001169681.2
dmCecropin_F	TCTTCGTTTTCGTCGCTCTC	NM_079849.4
dmCecropin_R	CTTGTTGAGCGATTCCCAGT	NM_079849.4
dmDefensin_F	GCCAGAAGCGAGCCACAT	NM_078948.3
dmDefensin_R	CGGTGTGGTTCCAGTTCCA	NM_078948.3
dmDiptericin_F	AGGTGTGGACCAGCGACAA	NM_057460.4
dmDiptericin_R	TGCTGTCCATATCCTCCATTCA	NM_057460.4
dmDrosocin_F	GCACAATGAAGTTCACCATCGT	NM_079020.5
dmDrosocin_R	CCACACCCATGGCAAAAAC	NM_079020.5
dmDrosomycin_F	CTCCGTGAGAACCTTTTCCA	NM_079177.4
dmDrosomycin_R	GTATCTTCCGGACAGGCAGT	NM_079177.4
dmMetchnikowin_F	GCTACATCAGTGCTGGCAGA	NM_079177.4
dmMetchnikowin_R	AATAAATTGGACCCGGTCT	NM_079177.4
dmActin_F	GCGTCGGTCAATTCAATCTT	NM_078901.3
dmActin_R	AAGCTGCAACCTCTTCGTCA	NM_078901.3
dmRpl32_F	ATGCTAAGCTGTCGCACAAATG	NM_079843.4
dmRpl32_R	GTTCGATCCGTAACCGATGT	NM_079843.4
***R. typhi* expression**		
**Oligonucleotide name**	**Oligonucleotide sequence**	**Reference**
adr1_F	CCACACCAGCACCAGCAAAA	(1)
adr1_R	GCACGGACAGTAGTTCAACC	(1)
ompB_F	TGGTATTACTGCTCAACAAGCT	(1)
ompB_R	CAGTAAAGTCTATTGATCCTACACC	(1)
***C. felis* expression**		
**Oligonucleotide name**	**Oligonucleotide sequence**	**NCBI accession number or Reference**
CfRel1_F	CCTCAATGGAATCTACTTCG	GAYP02012752
CfRel1_R	ACTTGTCTGTTGGTTGTTCC	GAYP02012752
CfRel2_F	ATCTAGCTGTGGCTGGAGGA	GAYP02012752
CfRel2_R	CTAAATGCAGGGGCGTATGT	GAYP02012752
CfImd_F	AAGCTCTCATGTCGTCTGAT	GAYP02008970
CfIMd_R	TTGTTTTGGGTCCAATCTAC	GAYP02008970
CfActin_F	CGGAGCCACACATTCTACAATGAACTC	GR302008.1
CfActin_R	CATGGTTAGCCTTTGGGTTCAA	GR302008.1
CfEf_F	TCGTACTGGCAAATCCACAG	(2)
CfEf_R	CATGTCACGGACAACGAAAC	(2)

REFERENCES

1. Gillespie JJ, Phan IQH, Scheib H, Subramanian S, Edwards TE, Lehman SS, Piitulainen H, Rahman MS, Rennoll-Bankert KE, Staker BL, Taira S, Stacy R, Myler PJ, Azad AF, Pulliainen AT. 2015. Structural Insight into How Bacteria Prevent Interference between Multiple Divergent Type IV Secretion Systems. MBio 6:e01867-15.
2. McIntosh CH, Baird J, Zinsen E, Woods DJ, Campbell EM, Bowman AS. 2016. Reference gene selection and RNA preservation protocol in the cat flea, *Ctenocephalides felis*, for gene expression studies. Parasitology 143:1532–1542.

Table S2 siRNA sequences for knockdown in *C. felis*.

siRNA name	siRNA sequence	NCBI accession number used to design siRNA
CfNeg siRNA 1	sic001-10nmol mission siRNA universal negative control #1 (Sigma)	
CfNeg siRNA 2	sic002-10nmol mission siRNA universal negative control #2 (Sigma)	
CfRelish siRNA 206_sense	GCA GAU AUG UAA UGG CUG A	GAYP02012752
CfRelish siRNA 206_antisense	UCA GCC AUU ACA UAU CUG C	GAYP02012752
CfRelish siRNA 676_sense	CCA UUG AGA CAA AGU GAA A	GAYP02012752
CfRelish siRNA 676_antisense	UUU CAC UUU GUC UCA AUG G	GAYP02012752
CfRelish siRNA 1277_sense	CAC CAG AAU UCA UCA GUA A	GAYP02012752
CfRelish siRNA 1277_antisense	UUA CUG AUG AAU UCU GGU G	GAYP02012752
CfImd siRNA 158_sense	CAG UUA GGC CAG CAA GAA A	GAYP02008970
CfImd siRNA 158_antisense	UUU CUU GCU GGC CUA ACU G	GAYP02008970
CfImd siRNA 208_sense	GAA CAG AAC AUU GGA GAA A	GAYP02008970
CfImd siRNA 208_antisense	UUU CUC CAA UGU UCU GUU C	GAYP02008970
CfImd siRNA 383_sense	CCU CAA UAC AGG AAG AUG A	GAYP02008970
CfImd siRNA 383_antisense	UCA UCU UCC UGU AUU GAG G	GAYP02008970

Table S3 Raw data used for analysis of the PCR array conducted in uninfected (Vero 1-5) and *R. typhi* infected (RT 1-5) KC167 cells

Well	Gene Symbol	Vero 1	Vero 2	Vero 3	Vero 4	Vero 5	Rt 1	Rt 2	Rt 3	Rt 4	Rt 5
A1	arr	25.52	25.52	24.79	26.47	26.07	27.34	25.19	26.22	26.29	25.47
A2	ato	34.83	34.83	32.16	32.77	37.47	35.2	33.63	39.14	35.39	30.98
A3	AttA	29.44	29.44	29.41	30.61	29.61	27.52	25.52	27.64	27.48	26.71
A4	AttB	30.26	30.26	28.68	30.46	29.56	27.67	25.91	28.44	27.46	26.41
A5	AttC	No Ct	No Ct	No Ct	37.96	38.14	33.71	33.57	34.5	No Ct	34.75
A6	AttD	34.06	34.06	31.97	30.68	31.1	31.5	29.06	30.89	30.18	28.56
A7	Axn	25.28	25.28	23.72	25.77	25.53	26.42	25.19	26.3	25.35	24.19
A8	bnl	23.49	23.49	23.4	23.95	23.93	25.02	23.26	24.05	23.94	23.12
A9	brk	26.01	26.01	25.4	26.96	26.83	27.42	25.75	27.05	26.54	25.99
A10	byn	No Ct	No Ct	No Ct	No Ct	No Ct	No Ct	38.98	33.43	37.82	30.33
A11	cact	22.25	22.25	21.47	22.9	22.95	23.53	21.62	22.73	22.69	22.12
A12	cathD	20.99	20.99	20.37	21.68	21.35	22.45	20.57	21.89	21.47	20.91
B1	CG10899	32.53	32.53	32.81	32.95	32.22	32.45	31.54	31.93	33.94	31.04
B2	CG10924	22.98	22.98	22.07	24.69	24.54	24.33	22.59	23.5	24.34	24.03
B3	CG2107	24.67	24.67	23.66	24.83	24.75	25.84	24.01	25.54	24.95	24.41
B4	CG3961	23.77	23.77	22.78	24.99	24.89	24.85	22.99	24.29	24.95	24.34
B5	CG8630	32.39	32.39	No Ct	No Ct	No Ct	34.94	No Ct	34.1	No Ct	33.42
B6	Csp	23.21	23.21	22.24	24.1	24.23	24.38	22.63	24.12	24.08	23.34
B7	CycD	25.26	25.26	24.03	25.45	24.95	26.32	24.43	25.83	25.32	24.1
B8	CycE	26.21	26.21	24.81	26.65	26.68	27.32	25.4	26.34	26.48	25.86
B9	CycG	20.82	20.82	20.55	21.76	21.63	22.6	20.64	21.83	21.73	20.88
B10	Cyp4e1	25.24	25.24	24.44	27.28	27.11	26.34	25.05	25.76	26.93	26.67
B11	dally	26.25	26.25	25.91	26.81	26.32	27.6	26.12	27.3	26.54	26.17
B12	Def	30.85	30.85	29.61	32.25	34.81	32.84	30.05	32.48	31.95	32.59
C1	Dl_Delta	30.34	30.34	29.25	30.36	30.51	30.8	31.04	32.47	30.69	29.71
C2	dm	23.39	23.39	22.62	23.78	23.76	25.27	22.91	23.96	23.57	22.54
C3	dpp	26.74	26.74	26.04	28.84	28.42	28.51	26.84	27.39	28.49	28.3
C4	Drs	27.42	27.42	26.73	29.05	28.34	28.78	27.06	27.93	29.19	27.95
C5	Ef1alpha48D	17.63	17.63	17.34	18.14	18.12	18.97	17.62	18.61	18.07	17.61
C6	ex	24.67	24.67	23.73	25.05	24.97	25.94	24.4	25.38	24.95	24.43
C7	Fatp	24.77	24.77	23.38	24.77	24.52	25.84	24.34	25.69	24.82	24.21
C8	fj	No Ct	No Ct	No Ct	No Ct	39.71	39.8	No Ct	No Ct	No Ct	32.64
C9	fkh	33.7	33.7	31.83	34.99	34.64	34.7	No Ct	34.91	35.8	33.77
C10	fng	28.98	28.98	27.18	28.78	29.34	30.03	28.49	29.61	28.98	28.62
C11	Fs	25.4	25.4	24.35	25.79	25.57	26.48	25.07	25.81	26.04	25.39
C12	fz	25.78	25.78	24.57	28.76	28.89	27.22	25.73	26.25	28.94	28.23
D1	Galphaf	26.47	26.47	25.39	29.52	29.48	27.19	25.63	26.49	30.29	27.48
D2	Gclm	24.46	24.46	23.54	24.51	24.3	25.39	23.62	24.98	24.55	23.89
D3	Glut1	24.56	24.56	23.77	25.52	25.38	25.81	24.45	25.24	25.29	24.78
D4	grn	34.33	34.33	No Ct	35.19	No Ct	35.23	35.42	33.58	34.99	32.21
D5	GstD1	18.04	18.04	17.75	18.86	18.57	19.28	17.79	18.77	18.77	18.41
D6	h	22.12	22.12	21.57	22.95	23.21	23.85	21.94	23.09	22.85	22.43

D7	hb	No Ct	No Ct	34.4	No Ct	35.4	36.66	39.95	34.14	No Ct	38.5
D8	Hey	No Ct	No Ct	37.18	No Ct	No Ct	No Ct	No Ct	No Ct	No Ct	35.86
D9	hkb	30.65	30.65	30.67	33.87	No Ct	32.85	31.06	30.84	No Ct	30.94
D10	Hsc70-1	33.55	33.55	No Ct	33.35	No Ct	34.35	No Ct	No Ct	32.99	35.59
D11	Hsp27	20.47	20.47	19.77	21.79	21.6	21.65	20.07	21.19	21.97	21.24
D12	iHog	25.74	25.74	25.41	26.13	26.1	27.68	25.48	26.66	26.27	25.77
E1	ImpL3	25.04	25.04	25.29	25.08	25.19	27.08	24.44	25.94	24.61	25.39
E2	Ir76a	25.12	25.12	24.59	25.69	25.3	26.57	24.29	25.9	25.56	24.85
E3	Keap1	23.38	23.38	22.53	24.22	24.13	24.77	22.9	23.96	23.97	23.6
E4	kibra	24.41	24.41	24.08	25.06	24.46	26.19	23.96	25.18	24.54	24.05
E5	mdy	21.21	21.21	20.53	22.47	22.13	22.59	20.76	21.9	22.23	21.69
E6	mirr	26.3	26.3	25.83	27.28	27.23	28.25	26.65	27.3	27.01	26.26
E7	Mtk	29.3	29.3	28.91	31.56	31.18	30.21	28.31	30.65	29.72	28.7
E8	PCNA	20.53	20.53	19.63	20.47	20.46	21.52	19.85	21	20.36	19.9
E9	N	26.13	26.13	25.11	26.43	26.24	27.55	26.17	26.7	26.13	25.4
E10	net	26.59	26.59	25.67	28.45	28.07	27.86	26.01	27.32	28.12	27.08
E11	nmo	28.72	28.72	28.14	29.96	29.82	30.79	28.28	28.63	28.97	28.61
E12	Odc1	No Ct	No Ct	No Ct	35.72	No Ct	No Ct	No Ct	35.61	No Ct	36.05
F1	Odc2	32.68	32.68	37.28	No Ct	No Ct	No Ct	33.15	No Ct	No Ct	34.93
F2	os	No Ct	No Ct	No Ct	34.64	33.6	No Ct	No Ct	34.31	32.57	37.86
F3	p53	21.4	21.4	20.72	21.29	21.16	22.43	20.65	21.92	21.51	20.81
F4	pan	23.55	23.55	22.9	24.91	25.02	25.02	23.12	24.22	24.77	24.02
F5	Pdk1	23.92	23.92	23.53	24.82	24.46	25.33	23.45	24.17	24.6	23.98
F6	ptc	27.4	27.4	27.08	28.91	28.92	29.58	27.3	27.62	28.47	28.13
F7	ref(2)P	22.25	22.25	21.54	22.87	22.77	23.65	22.13	22.7	22.64	22.47
F8	rept	24.61	24.61	23.44	24.56	24.6	25.89	24.12	24.97	24.14	23.79
F9	slmb	23.43	23.43	22.77	23.95	23.76	24.67	23.42	24.44	23.96	23.31
F10	smo	23.97	23.97	23.31	24.66	24.73	25.25	23.3	24.43	24.63	23.96
F11	Socs36E	24.24	24.24	23.74	24.55	24.15	25.8	24.07	25.12	24.28	24.05
F12	spi	20.73	20.73	19.95	21.56	21.34	22.09	19.98	21.3	21.73	20.98
G1	tgo	23.06	23.06	22.52	23.91	23.8	24.58	22.57	23.66	23.53	23.09
G2	th	21.9	21.9	21.59	23.03	22.76	23.54	21.77	22.94	23.03	22.21
G3	tll	No Ct	No Ct	No Ct	No Ct	33.54	35.05	34.06	34.64	No Ct	35.78
G4	Trx-2	21.26	21.26	20.12	21.49	21.34	22.25	20.59	21.81	21.93	21.29
G5	Tsp66E	28.84	28.84	27.36	28.54	29.16	29.4	28.09	29.03	28.61	27.91
G6	Ubx	No Ct	No Ct	No Ct	No Ct	32.42	34	32.18	38.86	No Ct	33.3
G7	upd2	29	29	27.97	27.85	27.43	30.08	29.3	29.63	27.67	26.66
G8	upd3	26.86	26.86	26.03	25.83	25.61	28.15	26.44	27.59	25.74	25.07
G9	vn	25.88	25.88	25.09	26.41	26.34	27.19	25.7	26.4	26.34	25.63
G10	wg	34.91	34.91	34.71	36.09	35.23	36.01	33.19	35.29	35.29	28.17
G11	Wnt4	33.06	33.06	36.66	38.76	34.7	35.05	No Ct	33.13	No Ct	30.59
G12	Wnt5	24.46	24.46	23.52	24.4	24.31	26	23.99	25.12	24.42	23.65
H1	Act42A	20.91	20.91	19.98	21.01	20.95	21.82	20.35	21.48	20.45	19.4
H2	Gapdh1	21.01	21.01	20.2	21.33	21.27	22.09	20.14	21.63	21.28	20.28
H3	RpL32	17.4	17.4	16.69	17.81	17.73	17.99	16.91	17.98	17.93	17.6
H4	SdhA	23.06	23.06	22.34	23.37	23.27	24.58	22.47	23.74	23.07	22.77

H5	Tbp	23.86	23.86	23.61	24.74	24.26	25.31	23.31	24.66	24.39	23.94
H6	DGDC	34.1	34.1	No Ct	No Ct	No Ct	No Ct	No Ct	No Ct	No Ct	34.83
H7	RTC	20.64	20.64	19.52	20.59	20.15	21.42	20.45	20.99	20.27	20.16
H8	RTC	20.74	20.74	19.63	20.7	20.2	21.46	20.42	21.08	20.28	20.21
H9	RTC	20.63	20.63	19.47	20.46	20.14	21.42	20.38	21.07	20.25	20.14
H10	PPC	17.66	17.66	16.87	18.36	18.04	18.42	17.87	18.26	18.05	17.82
H11	PPC	17.81	17.81	16.92	18.36	18.03	18.56	17.92	18.2	17.97	17.82
H12	PPC	17.82	17.82	16.82	18.4	18.08	18.56	18.01	18.09	18.11	17.79
